# Fingolimod-associated severe bilateral cystoid macular edema

**DOI:** 10.1016/j.ajoc.2022.101553

**Published:** 2022-04-22

**Authors:** Hideki Fukuoka, Kentaro Kojima, Ayako Iwama, Takahiro Okumura, Chie Sotozono

**Affiliations:** Department of Ophthalmology, Kyoto Prefectural University of Medicine, Kyoto, Japan

**Keywords:** Bilateral macular edema, Fingolimod, Cataract surgery

## Abstract

**Purpose:**

To report a rare case in which a patient undergoing long-term oral fingolimod treatment for multiple sclerosis (MS) developed late-onset severe bilateral cystoid macular edema (ME) at 3-weeks post cataract surgery.

**Observations:**

This study involved a 61-year-old female undergoing long-term oral fingolimod treatment for MS in whom at 4-years post initiation of treatment and the treatment being tapered to a 0.5 mg twice-weekly dose severe bilateral cystoid ME occurred at 3-weeks post cataract surgery. Although the patient was administered the proper treatments for pseudophakic ME, including a 20-mg sub-Tenon's-capsule triamcinolone acetonide injection, it took 13 months for the ME to ultimately resolve with continued oral fingolimod treatment.

**Conclusions and importance:**

The findings in this study show that severe bilateral cystoid ME can occur even several years after initiating fingolimod treatment, thus indicating that detailed follow-up is necessary post cataract surgery.

## Highlights

• Severe cystoid ME can occur even several years after initiating fingolimod treatment.
• Fingolimod-associated ME post cataract surgery did not resolve with STTA in this case.
• It took 13 months for the ME to ultimately resolve with continued oral fingolimod treatment.

## 1 Introduction

Multiple sclerosis (MS) is a chronic disease of the central nervous system (CNS) characterized by focal areas of demyelination in the CNS white matter with secondary neuroaxonal degeneration. Reportedly, the clinical manifestations of MS vary depending on the location of demyelinating brain lesions.^1^ Fingolimod is a sphingosine-1-phosphate receptor modulator that indirectly antagonizes the function of the receptor, thus leading to lymphocyte sequestration in the lymph nodes and a reduction of their presence in the CNS, and it is currently approved for the treatment of relapsing-remitting MS. However, magnetic resonance imaging (MRI) findings in a clinical trial demonstrated that although treatment with fingolimod results in a reduction of MS relapses and disability progression, brain activity and volume loss can occur.^2^

It has been reported that fingolimod-associated macular edema (ME) was observed in 0.4% of the MS patients treated with 0.5 mg fingolimod (the FDA-approved dose) and in 1.0% of those treated with 1.25 mg fingolimod.^3^ In that study, the findings showed that the onset of ME occurred within approximately 3 months after the treatment was initiated. However, the ME typically resolved in the majority of the patients in that study soon after the cessation of treatment.

Herein, we report a rare case of severe bilateral cystoid ME that occurred in an MS patient many years after the initiation of treatment with oral fingolimod.

## 2 Case report

This study involved a 61-year-old Asian female who was diagnosed with MS in 2007. For treatment, she underwent interferon-beta injection until April 2014. During that treatment course, she experienced hypertension, peripheral neuropathy, and suspected Sjögren's syndrome, and was taking 500 μg of mecobalamin (a coenzyme form of vitamin B12) 3-times daily, 200 mg of tocopherol nicotinate (a fat soluble vitamin) 3-times daily, and 5 mg of baclofen (an oral medication for MS-related spastic movement) 2-times daily. Moreover, she experienced three episodes of optic neuritis.

In October 2014, the findings of a cranial MRI examination revealed several new brain lesions, and treatment with oral fingolimod was initiated (0.5 mg, 4-times daily). For 4 years following the initiation of the fingolimod therapy, no treatment-related adverse effects, including the development of ME, were observed.

Upon initial presentation at our department, our examinations revealed that she had age-related bilateral cataracts and a visual acuity (VA) of 8/20 OD and 6/20 OS. On July 10, 2018, she underwent cataract surgery in her left eye, followed by cataract surgery in her right eye 2-weeks later. Both operations were successful, and no surgical complications were observed. Since her MS was deemed stable, she was then treated with a twice-weekly (i.e., every Wednesday and Sunday) administration of 0.5 mg fingolimod. Moreover, during the postoperative treatment course, she underwent a 4-times-daily administration of 1.5% levofloxacin eye drops and 0.1% betamethasone eye drops. At 1-week postoperative, her spectacle-corrected VA was found to have improved to 16/20 OD and 10/20 OS. At 3-weeks postoperative, she reported blurred vision in her left eye, yet with no ocular or eye-movement-related pain. Two weeks later, she reported the occurrence of that same problem in her right eye. An ophthalmic examination showed that her VA had decreased to 6/20 OD and 4/20 OS, and a subsequent optical coherence tomography (OCT) examination revealed severe bilateral cystoid ME. Thus, we initiated treatment with an additional 4-times-daily administration of bromfenac eye drops and a 6-times-daily administration of 0.1% betamethasone eye drops.

Due to the development of the severe bilateral cystoid ME, the patient was immediately referred to the Department of Neurology at our institution, as we were unable to determine whether the ME was fingolimod-related or pseudophakic cystoid ME. Upon examination, it was determined that the patient should continue treatment with oral fingolimod, as the findings in an FDA report showed that cessation of fingolimod can sometimes exacerbate MS.^4^

**Fig. 1:**
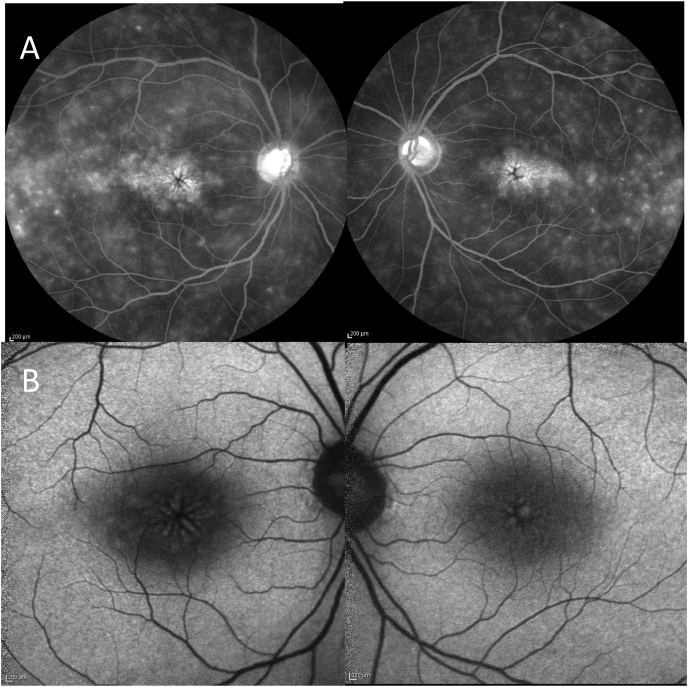
Fundus fluorescein angiography (FFA) and fundus autofluorescence (FAF) images taken at 3 months after the occurrence of macular edema (ME). (A) FFA image of both eyes in the late phase showing leakage into the cystoid spaces distributed radially in the outer plexiform layer (Henle's layer) forming the classic petaloid staining pattern. (B) FAF image of both eyes showing mild hyperautofluorescence petaloid pattern in the fovea. Left columns: right eye, right columns: left eye.

**Fig. 2:**
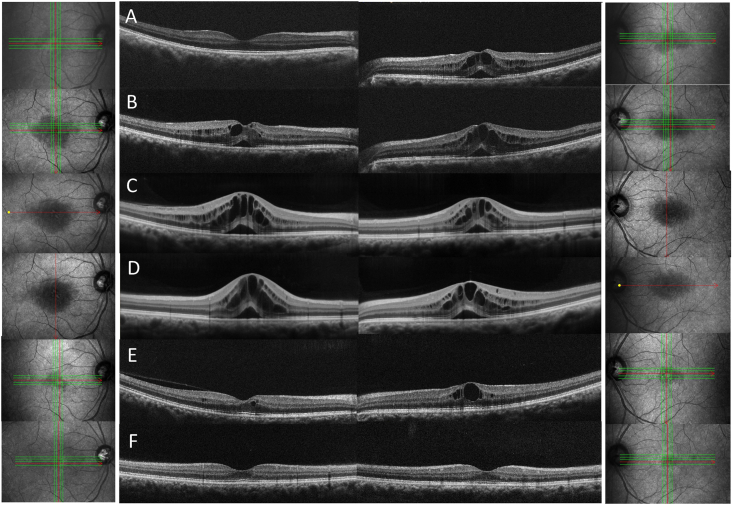
Sequential optical coherence tomography (OCT) images showing bilateral ME (A–E). Left columns: right eye, right columns: left eye. (A) OCT image showing the development of ME in the patient's left eye at 3-weeks post cataract surgery. No ME in right eye at 1 week after cataract surgery. (B) OCT image showing the development of ME in the patient's right eye at 3-weeks post cataract surgery. (C) OCT image showing that the ME in both eyes had worsened at 3-months post cataract surgery. (D) OCT image showing that treatment with a sub-Tenon's 20 mg triamcinolone acetonide injection in the patient's left eye was ineffective. (E) OCT image showing a decrease of ME at 1-year post cataract surgery. (F) OCT image showing no bilateral ME at 13-months post cataract surgery.

In October 2018, the patient underwent treatment with a 20-mg sub-Tenon's capsule triamcinolone acetonide injection (STTA) in her left eye. However, post STTA, the ME did not improve. At 3 months after ME occurred, fundus fluorescein angiography and fundus autofluorescence were performed (Fig. 1). OCT findings revealed that bilateral ME progressed at 5–9 months post onset, yet decreased at 10–12 months post onset. A follow-up examination performed at 13-months post cataract surgery revealed no bilateral persistent ME, as well as that her VA was 4/20 OD and 3/20 OS (Fig. 2).

## 3 Discussion

ME is defined as an abnormal increase of fluid volume and proteins that collect on or under the macula and a major cause of visual impairment in the course of metabolic, vascular, and inflammatory retinal diseases that ultimately leads to the loss of central vision. Diabetes, retinal vein occlusion, uveitis, age-related macular degeneration, and ocular surgeries, including cataract surgery, are well-known risk factors for development of pseudophakic ME.^5^

The FDA guidelines recommend that ophthalmic examinations of the macula should be performed at 3-months after the initiation of fingolimod treatment, as almost all cases of fingolimod-associated ME occur at or around that time point. In our present case, the findings suggest that cataract surgery may have possibly triggered the development of ME following fingolimod treatment, as bilateral pseudophakic ME after cataract surgery is relatively rare and our patient still has bilateral persistent ME despite undergoing appropriate treatment for pseudophakic ME. Moreover, she had no potential risk factors for developing pseudophakic ME, such as diabetes and uveitis, etc.

Although previous studies have reported fingolimod-associated ME post cataract surgery being resolved with STTA^6^ and fingolimod-associated ME treated with intravitreal injection and continued fingolimod use^7^ (as well as fingolimod discontinuation without topical eye-drop instillation^8^), to the best of our knowledge, this is the first reported case of bilateral ME post cataract surgery that spontaneously resolved at 13-months postoperative following initial treatment with fingolimod at the current lower FDA-approved dose and continued fingolimod use.

## 4 Conclusion

The findings in this study revealed that detailed follow-up is necessary after eye surgery, as we discovered that severe bilateral cystoid ME can occur many years after initiating fingolimod treatment post cataract surgery since that treatment may be one of the risk factors for the development of pseudophakic ME.

## Patient consent

Consent to publish this case report has been obtained from the patient in writing. This case report does not contain any personal identifying information.

## Funding/support

No funding or grant support

## Authorship

All authors attest that they meet the current ICMJE criteria for Authorship.

## Author contributions

Hideki Fukuoka: Conceptualization, Writing- Original draft preparation, Kentaro Kojima: Investigation, Ayako Iwama: Data curation, Takahiro Okumura: Data curation, Chie Sotozono: Supervision, Writing- Reviewing and Editing.

## Declaration of competing interest

No conflicting relationship exists for any author.
